# *GATA3* frameshift mutation promotes tumor growth in human luminal breast cancer cells and induces transcriptional changes seen in primary *GATA3* mutant breast cancers

**DOI:** 10.18632/oncotarget.21910

**Published:** 2017-10-20

**Authors:** John P. Gustin, Jernelle Miller, Mina Farag, D. Marc Rosen, Matthew Thomas, Robert B. Scharpf, Josh Lauring

**Affiliations:** ^1^ Sidney Kimmel Comprehensive Cancer Center at Johns Hopkins, Baltimore, MD, USA

**Keywords:** GATA3, mutation, breast cancer, luminal

## Abstract

The *GATA3* transcription factor is one of the most frequently mutated genes in breast cancer. Heterozygous mutations, mostly frameshifts, are seen in 15% of estrogen receptor positive breast cancers, the subtype in which these mutations are almost exclusively found. Mouse studies have shown that Gata3 is critical for breast development and that *GATA3* gene dosage affects breast tumor progression. Human patient data have shown that high Gata3 expression, a feature of luminal subtype breast cancers, is associated with a better prognosis. Although the frequency of *GATA3* mutation suggests an important role in breast cancer development or progression, there is little understanding of how mutations in *GATA3* affect its function in luminal breast epithelial cells and what gene expression changes result as a consequence of the mutations. Here, using gene editing, we have created two sets of isogenic human luminal breast cancer cell lines with and without a hotspot truncating *GATA3* mutation. *GATA3* mutation enhanced tumor growth *in vivo* but did not affect sensitivity to clinically used hormonal therapies or chemotherapeutic agents. We identified genes with upregulated and downregulated expression in *GATA3* mutant cells, a subset of which was concordantly differentially expressed in *GATA3* mutant primary luminal breast cancers. Addback of mutant *GATA3* recapitulated mutation-specific gene expression changes and enhanced soft agar colony formation, suggesting a gain of function for the mutant protein.

## INTRODUCTION

Heterozygous mutations in the *GATA3* gene, encoding a transcription factor crucial for breast development, occur in 15% of estrogen receptor-positive (ER+), or luminal-type, breast cancers [1, 2]. Mutations in *GATA3* are heterogeneous, but almost all of the mutations affect splice sites or are insertions/deletions (indels) that result in translational frameshifts. Many of these mutations result in premature termination of translation and truncated proteins lacking all or part of the second zinc finger, which mediates DNA binding [3]. Another cluster of mutations occurs near the C-terminus of Gata3, and it is not clear whether these mutations affect Gata3 function in the same manner as truncating mutations. Some of the breast cancer-associated truncating mutations cluster in the same region as mutations in the HDR syndrome (hypoparathyroidism, sensorineural deafness, and renal insufficiency), an autosomal dominant disorder ascribed to Gata3 haploinsufficiency [4]. However, mutant *GATA3* transcripts and proteins are highly expressed, and the mutational bias toward the distal part of the protein suggests that these mutations do not cause a simple loss of function.

Gata3 expression is highest in hormone receptor-positive breast cancers. Several studies have shown that Gata3 expression correlates with a better prognosis, which is not surprising given the tight correlation between Gata3 expression and ER expression (> 90% co-expression) [5–9]. Since *GATA3* mutations in most cases examined do not lead to loss of transcript or protein, they are not identified by prognostic studies using gene expression microarrays or immunohistochemistry. The METABRIC study reported that *GATA3* mutant tumors have a favorable prognosis compared to *GATA3* wild type ER+ breast cancers [1]. However, the prevalence of GATA3 mutations in a population of treatment refractory metastatic breast cancers was identical to that reported in primary tumors (12%), suggesting that GATA3 mutant tumors are not especially favorable [10].

Studies using human breast cancer cell lines show that Gata3 co-regulates certain genes with the estrogen receptor alpha (ERα) and that there may be reciprocal regulation between Gata3 and ERα [11, 12]. A gene expression signature enriched for genes induced by both estrogen and Gata3 defined a good prognosis subgroup of breast cancer patients, however Gata3-regulated genes were defined as those induced by overexpression of Gata3 in HEK-293 kidney cells, rather than in breast epithelial cells [13]. Several Gata3 target genes have been proposed, including *CCND1*, *CDKN2C*, *MUC1*, and *ESR1* [14–16], however the target genes affected by Gata3 mutations in human breast cancers have not been elucidated.

Prior work by others has demonstrated a range of phenotypes with ectopic Gata3 overexpression or knockdown. Studies examining the effect of expressing wild type Gata3 in ER-negative cell lines such as MDA-MB-231 have shown that Gata3 favors expression of epithelial over mesenchymal markers and negatively regulates breast cancer metastasis [17–21]. However, such studies, while suggestive, do not address the function of Gata3 in the luminal breast cell types where it is highly expressed and frequently mutated. Ectopic overexpression or gene knockdown do not always recapitulate the phenotypes generated by physiologic expression of cancer-associated mutations using gene editing [22–24]. Here, we have utilized gene editing in human ER+ breast cancer cell lines to identify phenotypes and transcriptional targets dependent on mutant *GATA3*.

## RESULTS

### Isogenic human breast cancer cell line models to study effects of *GATA3* mutations

**Figure 1:**
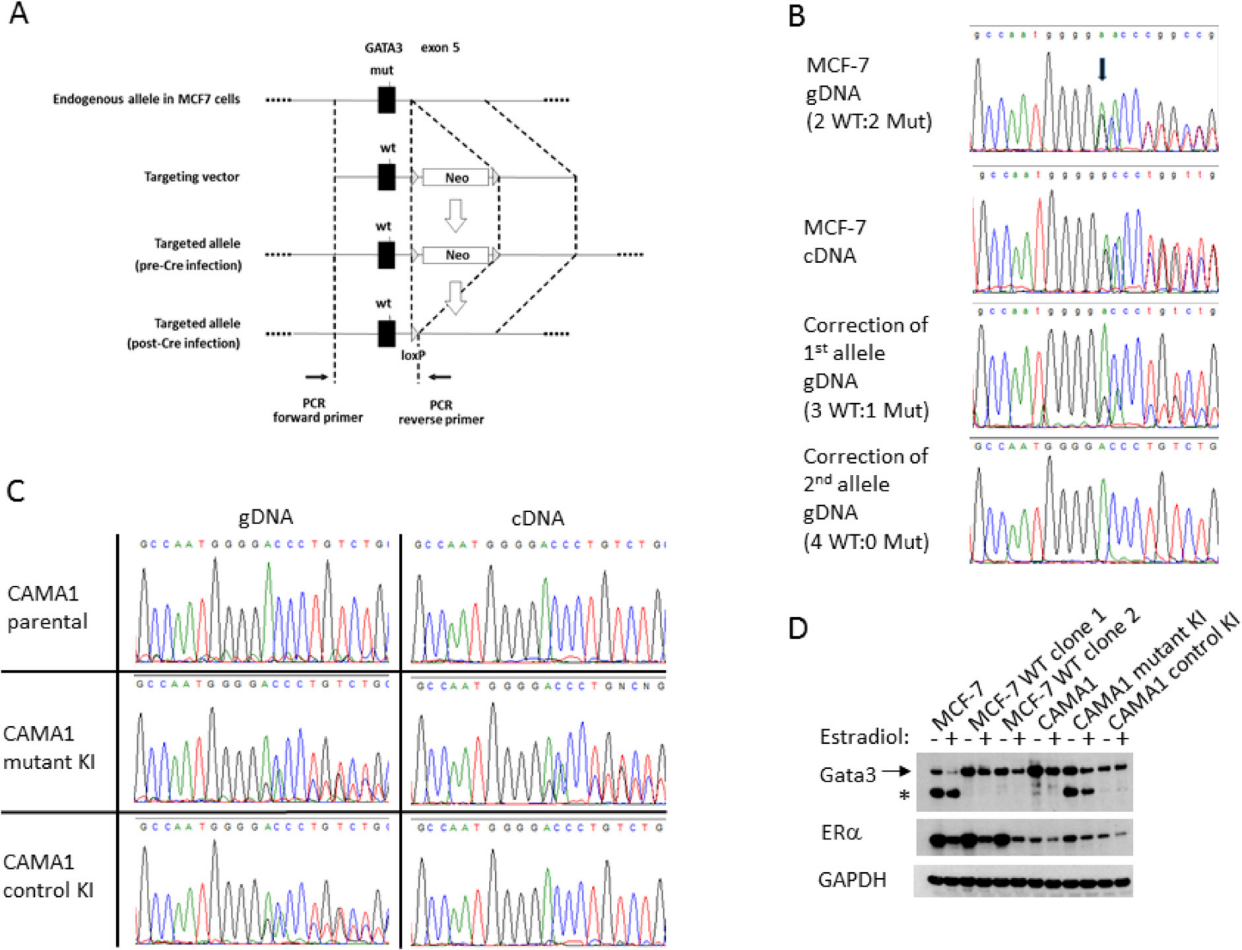
Correction of the *GATA3* D336Gfs*17 mutation in MCF-7 and knock-in of *GATA3* D336Gfs*17 in CAMA1 cells using gene targeting (**A**) Schematic of the gene targeting construct used for the first allele. (**B**) Sequencing traces showing correction of first and second mutant *GATA3* alleles in MCF-7. A single representative clone is shown for each step. Arrow indicates start of frameshift. (**C**) Sequencing trace of gDNA and cDNA showing knock-in of mutant *GATA3* alleles in two CAMA1 clones, one of which lost expression of the mutant *GATA3* transcript (“control KI”). (**D**) Western blot showing full length (arrow) and truncated (asterisk) Gata3 proteins in parental MCF-7 cells, two *GATA3* wild type corrected clones, parental CAMA1 cells, *GATA3* mutant knock-in (KI) clone, and control KI clone. Cells were cultured in the presence or absence of 17-β-estradiol. Estrogen receptor (ERα) and GAPDH proteins are shown for comparison.

In order to study the functional consequences of *GATA3* mutations in a human breast cancer system, we utilized the MCF-7 cell line, widely used as a representative model for ER+, luminal-type breast cancer. MCF-7 cells have a naturally occurring *GATA3* mutation, a G insertion in exon 5, leading to a frameshift and premature truncation of the translated polypeptide (D336Gfs*17, Figure 1) [4]. This mutation occurs in the second zinc finger of the Gata3 protein, and such mutations have been shown to disrupt binding to GATA motifs in DNA [3, 25]. This mutation is a recurrent hotspot in primary human breast cancers, having been reported 16 times in the TCGA and METABRIC datasets. Therefore, MCF-7 is a relevant model to understand the functional consequences of *GATA3* truncating mutations that occur almost exclusively in ER+ breast cancers. We confirmed the presence of the *GATA3* mutation in our MCF-7 cells by sequencing genomic DNA as well as cDNA. The mutation is heterozygous and equally expressed at the mRNA level with the wild type allele (Figure 1B). Western blotting confirmed the presence of full length and truncated forms of the Gata3 protein of the expected sizes (Figure 1D). It is interesting to note that the truncated Gata3 polypeptide is more abundant than the wild type protein, despite equal transcription, likely reflecting increased protein stability.

We designed a rAAV gene targeting vector to replace the mutant *GATA3* exon 5 with a wild type copy (Figure 1A). We obtained three targeted clones. Sequencing revealed replacement of the mutant allele with wild type sequence (Figure 1B); however, as suggested by published copy number data, MCF-7 appears to have four copies of chromosome 10, with two wild type and two mutant *GATA3* alleles. Thus, the first round of correction left three wild type alleles and one mutant allele. We therefore designed a second targeting construct with *GATA3* exon 5 in the 3’ homology arm, to minimize retargeting events. Two homozygous wild type *GATA3* MCF-7 (hereafter referred to as *GATA3* WT) clones were derived, each from an independent first-allele-targeted clone. Sequencing of genomic DNA and cDNA confirmed the complete absence of the G insertion mutation (Figure 1B and data not shown), and western blotting demonstrated full length Gata3 protein only. *GATA3* WT clones showed similar levels of full length Gata3 and ERα to parental MCF-7 cells (Figure 1D). Successful recovery of fully wild type *GATA3* clones shows that MCF-7 cells do not require mutant Gata3 for survival.

We developed a second model using CAMA1, another ER+ breast cancer cell line, which is *GATA3* wild type. We used the same gene targeting strategy to introduce the MCF-7 derived D336Gfs*17 mutation on a single allele in CAMA1 cells. Sanger sequencing shows a less than 1:1 ratio of wild type and mutant *GATA3* alleles in the targeted CAMA1 clones (Figure 1C). Droplet digital PCR copy number analysis shows that the CAMA-1 cells likely have four copies of the *GATA3* locus, like MCF-7 (data not shown). Thus, the CAMA1 *GATA3* mutant knock-in clones do not fully recapitulate the allelic ratio of MCF-7 and most primary breast cancers. Initially two mutant knock-in clones were obtained. However, the second *GATA3* mutant CAMA1 clone lost expression of the mutant allele, presumably through epigenetic downregulation, since the mutant allelic ratio remained approximately 1:3 in genomic DNA, whereas the mutant transcript and protein were barely detectable by RT-PCR and western blotting (Figure 1C and 1D). This second clone was therefore used as a wild type control (hereafter referred to as “Control KI”), having been through the same gene editing process as the GATA3 mutant-expressing clone.

### Growth and drug sensitivity of *GATA3* mutant and wild type cells

**Figure 2:**
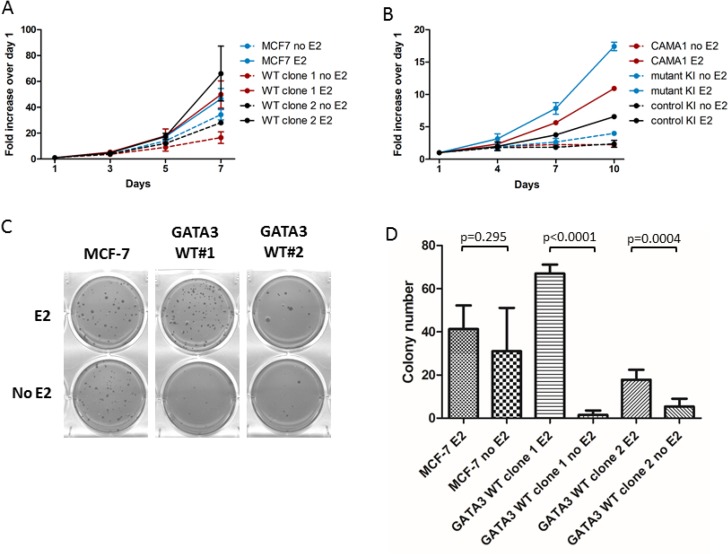
Growth comparison of MCF-7 and *GATA3* wild type derivatives (**A**) Proliferation of parental MCF-7 and *GATA3* wild type derivative clones in the presence and absence of estrogen (E2). (**B**) Proliferation of parental CAMA1, *GATA3* mutant knock-in, and control knock-in cells in the presence and absence of estrogen. (**C**) Colony formation in soft agar by MCF-7 and *GATA3* wild type derivative clones in the presence and absence of estrogen. (**D**) Quantification of soft agar colonies (average of two experiments). Means and standard deviations are shown. *P* values are from unpaired *t*-tests for the comparisons shown.

In cell culture, the two *GATA3* WT clones proliferated with similar kinetics to parental MCF-7 cells overall, whether grown in low-serum (0.5% CD-FBS) or in the presence of 17-β-estradiol. Significant differences were only observed between MCF-7 and WT clone 1 in the absence of estrogen and between MCF-7 and WT clone 2 in the presence of estrogen (adjusted *p* < 0.01 for each comparison, Figure 2A). *GATA3* mutant knock-in CAMA1, however, grew faster compared to *GATA3* wild type parental CAMA1 or control knock-in cells cells in low serum and in the presence of estrogen (*p* < 0.001 for all comparisons, Figure 2B). In soft agar, parental MCF-7 cells formed colonies equally well in the presence or absence of estrogen (*P* value non-significant), whereas both *GATA3* WT clones formed significantly more colonies in the presence of estrogen (Figure 2C and 2D). Thus, *GATA3* mutation appears to render MCF-7 cells less dependent on estrogen for anchorage-independent growth. Of note, *GATA3* mutation did not affect the estrogen-induced downregulation of ERα, and both wild type and truncated Gata3 proteins were downregulated concordantly in response to estrogen (Figure 1D). Attempts to grow CAMA1 cells in soft agar were unsuccessful.

**Figure 3:**
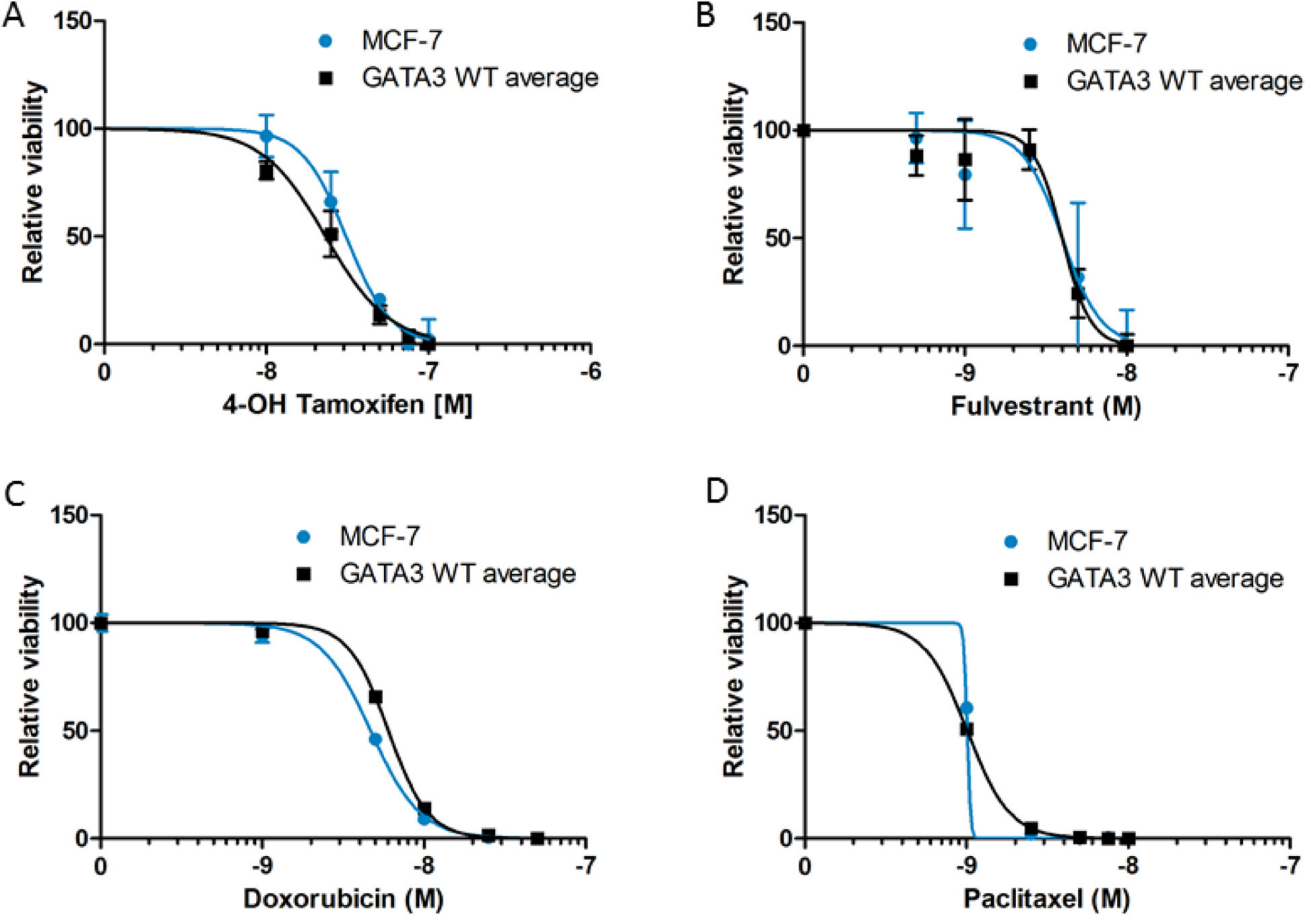
Drug sensitivity of MCF-7 and *GATA3* wild type derivative clones to 4-OH tamoxifen (**A**), fulvestrant (**B**), doxorubicin (**C**), and paclitaxel (**D**). Cell viability relative to untreated control is shown for each cell line. Graphs depict curve fit of the data using nonlinear regression for MCF-7 versus the average of the two wild type clones.

**Figure 4:**
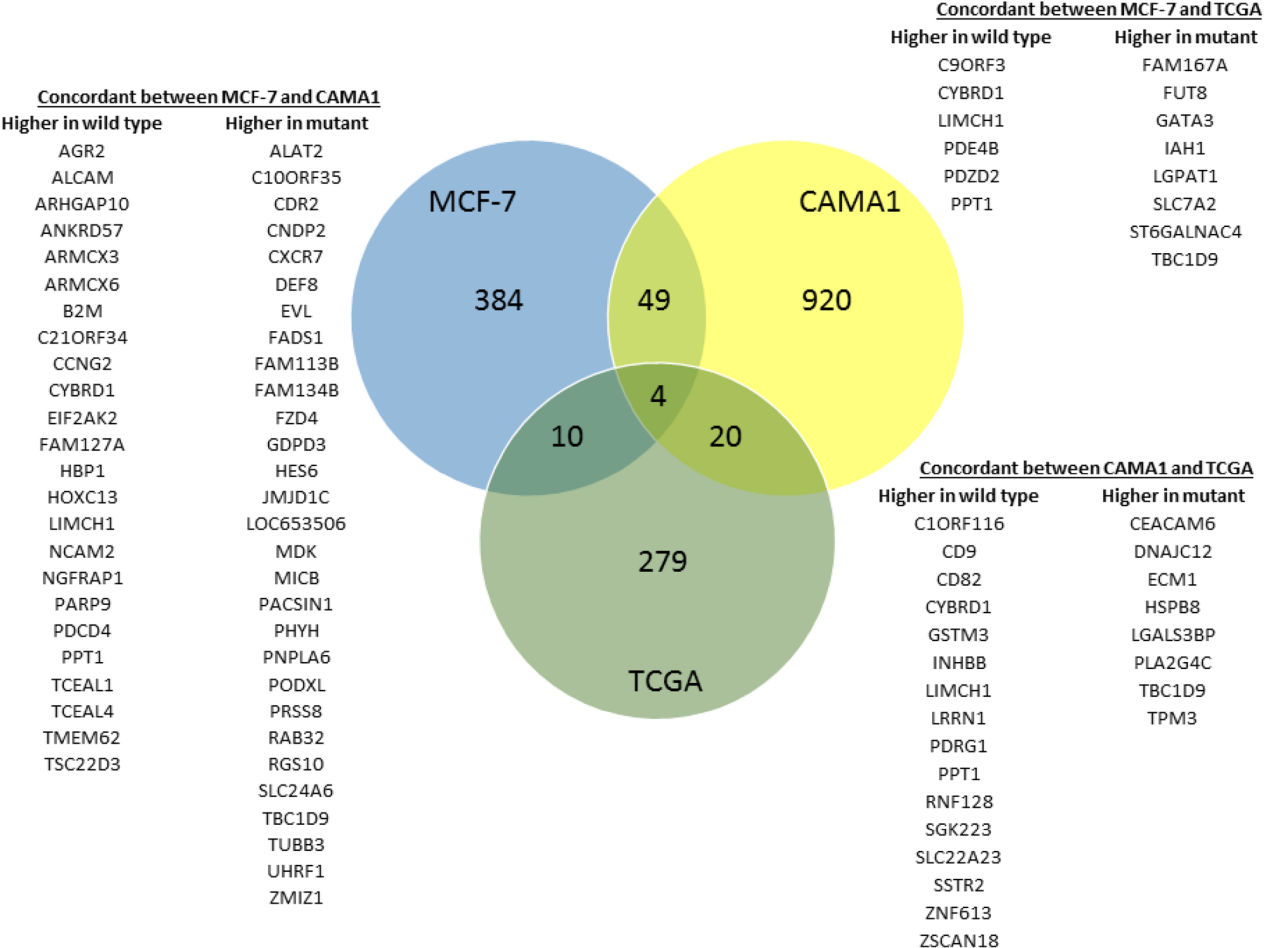
Differential gene expression related to *GATA3* mutation Venn diagram showing overlap in differentially expressed genes in the isogenic MCF-7 and CAMA1 cell line panels and 594 ER + breast cancers from the TCGA dataset. Overlap between gene lists from MCF-7, CAMA1, and TCGA are all greater than expected by chance and statistically significant by hypergeometric probability (*P* < 0.004 for MCF-7/TCGA, *P* < 0.007 for CAMA1/TCGA, and *P* < 7 × 10–11 for MCF-7/CAMA1).

Because Gata3 has been implicated in the co-regulation of ER target genes and because of the differential soft agar growth response in the absence of estrogen, we hypothesized that parental MCF-7 cells with mutant Gata3 would be more resistant to hormonal therapies. Cells were grown in 1 nM estradiol and treated with a range of concentrations of the active metabolite 4-OH tamoxifen (Figure 3A) or the selective estrogen degrader fulvestrant (Figure 3B). There was no significant difference in the sensitivity of the cells to hormonal therapy according to *GATA3* mutation status (*p* = ns by ANOVA). The cells also exhibited similar sensitivity to the chemotherapeutic drugs doxorubicin (although the IC50 difference of 1.4nM was statistically significant, *p* < 0.0001, Figure 3C) and paclitaxel (Figure 3D). The similar sensitivity to paclitaxel is notable, as the *GATA3* mutant MCF-7 cells showed higher transcript levels of *TUBB3*, encoding the beta 3 isoform of tubulin, which has been associated in some studies with paclitaxel resistance (Figure 4 and Supplementary Table 1) [26].

### Transcriptional consequences of *GATA3* mutations

Since Gata3 is a transcription factor and a master regulator of breast development, we hypothesized that *GATA3* mutation would alter transcription of downstream target genes. Therefore, we performed whole-genome gene expression analyses comparing MCF-7 parental and *GATA3* WT cells and CAMA1 parental and *GATA3* mutant cells. Four hundred forty-seven genes were differentially expressed in MCF-7 cells according to *GATA3* mutation status at a Benjamini-Hochberg adjusted *p* value of < 0.05 (Supplementary Table 1). In CAMA1 cells, 993 genes were differentially expressed at 1.25-fold or greater between parental CAMA1 and the *GATA3* mutant knock-in cells. Given the large number of genes in the genomic platforms and the limited replicates for each clone, we sought to identify concordantly over-expressed genes in the two cell lines, as we hypothesize that expression of bona-fide targets of GATA3 are more likely to be similarly effected in both cell lines. We defined genes with concordant differential expression in both cell lines by statistically significant moderated t (MCF-7 mutant versus wild type) and 1.25-fold change in expression (CAMA1 parental versus GATA3 mutant knock-in clone). Fifty-three genes were concordantly differentially expressed according to *GATA3* mutation status between the MCF-7 and CAMA1 cell line panels, (*p* < 7 × 10^−11^ by hypergeometric probability test, Figure 4).

In order to understand the relevance of the gene expression changes we observed in our isogenic cell lines to primary human breast cancers, we examined gene expression in the TCGA breast cancer dataset. The TCGA dataset contains 594 primary ER+ breast cancers with RNAseq and mutation data, of which 84 (14%) have *GATA3* mutations. 313 genes were differentially expressed in *GATA3* mutant versus wild type cases at a Benjamini-Hochberg q value of < 0.01 (Supplementary Table 1). Of these 313 genes, 14 are concordantly differentially expressed in the MCF-7 isogenic cell lines and 24 in the CAMA1 cell lines (*p* < 0.004 and *p* < 0.007, respectively, by hypergeometric probability test, Figure 4). Four genes showed concordant differential gene expression in all three datasets: *CYBRD1*, *PPT1*, *LIMCH1* and *TBC1D9*.

**Figure 5:**
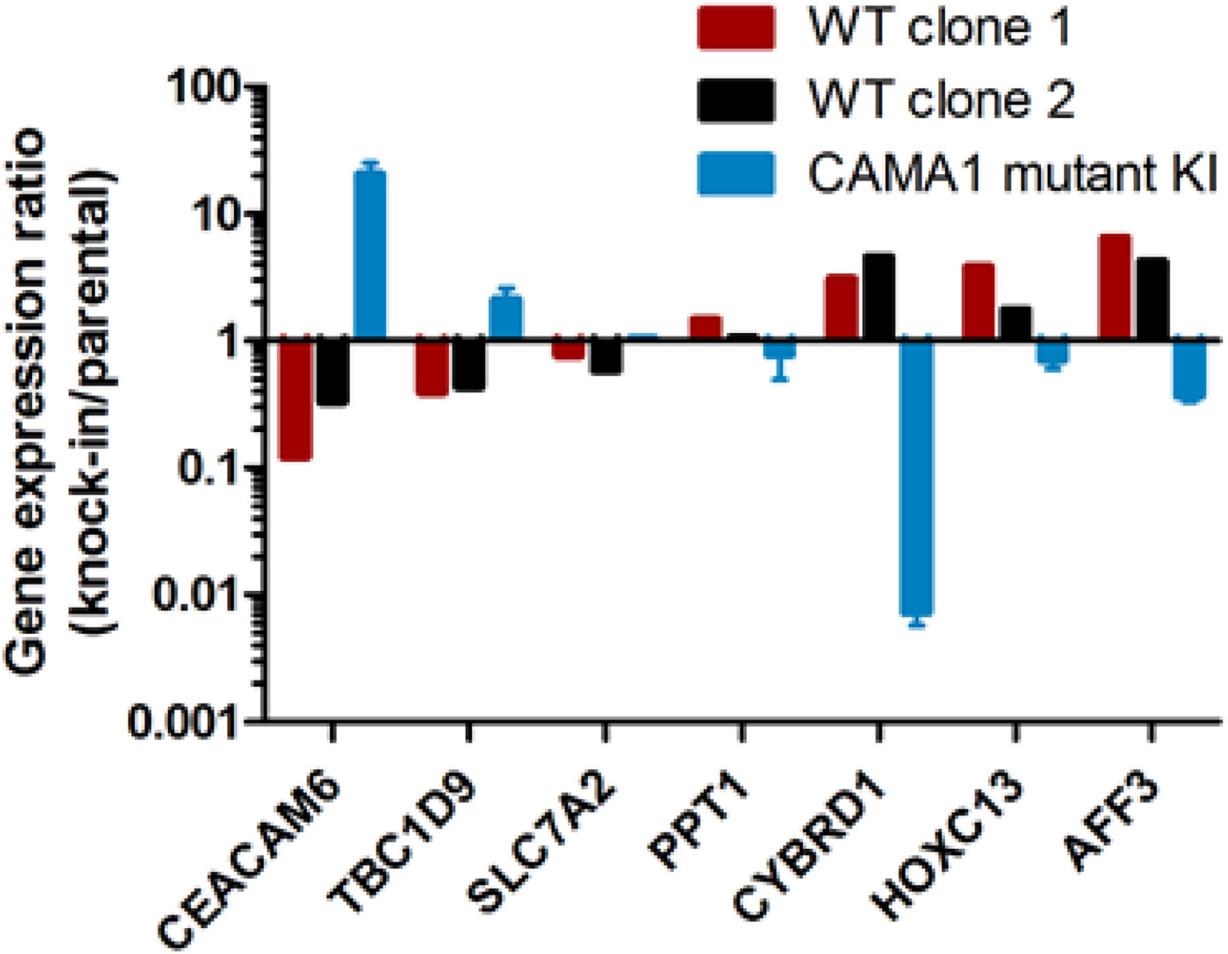
Gene expression changes effected by mutant Gata3 Quantitative RT-PCR for selected genes differentially expressed by microarray analysis. Gene expression for gene-edited clones is depicted as fold-change relative to the level in the parental cell line (X-axis). Bars are in opposing directions because *GATA3* knock-in was wild type for MCF-7 and mutant for CAMA1.

**Figure 6:**
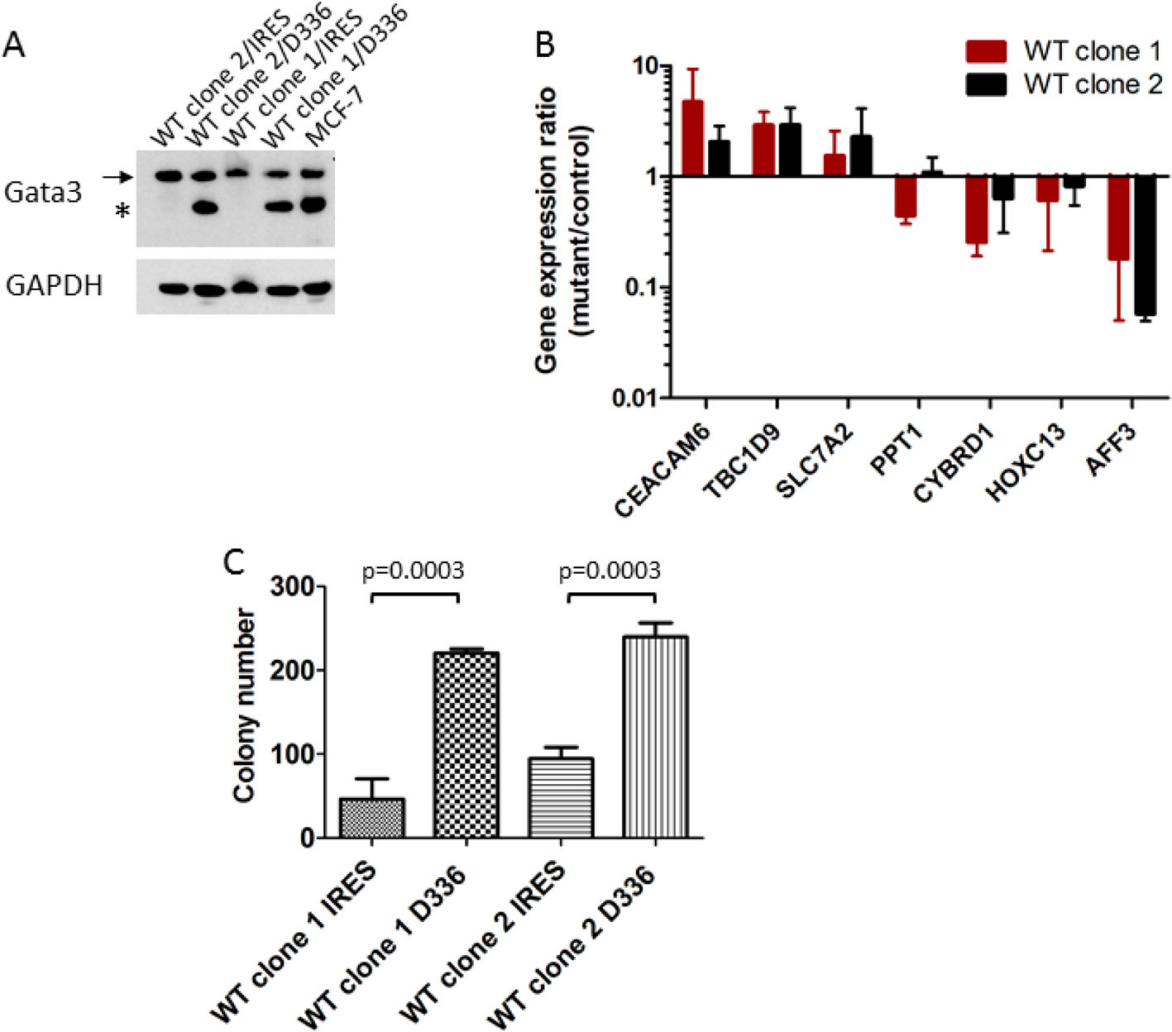
Add-back of mutant GATA3 supports a gain of function (**A**) Re-expression of Gata3 D336Gfs*17 mutant protein in MCF-7-derived GATA3 wild type clones (“D336”). Western blot shows wild type (arrow) and mutant (asterisk) Gata3 proteins in transfected clones and MCF-7. (**B**) Quantitative RT-PCR for candidate Gata3 target genes. Gene expression ratio (fold change) for each clone is shown (D336Gfs*17 mutant/IRES vector control). Means and standard deviations from four experiments are shown. (**C**) Soft agar colony formation of MCF-7 derived GATA3 wild type clones transfected with vector (IRES) or GATA3 D336Gfs*17 mutant (D336). Means and standard deviations from three replicate wells are shown. *P* = 0.0003 by *t* test for difference between IRES and D336 for each clone.

Differential expression of several of these genes was verified in the MCF-7 and CAMA1 cell line panels by qRT-PCR, including some interrogated genes that did not show consistent changes by microarray between the two cell lines (Figure 5). Expression of *TBC1D9* and *CEACAM6* was higher in *GATA3* mutant MCF-7 cells, *GATA3* mutant CAMA1 cells, and primary *GATA3* mutant breast cancer cells, whereas expression of *PPT1*, *CYBRD1*, *HOXC13*, and *AFF3* was higher in *GATA3* wild type cells (Figures 4 and 5). Importantly, the gene targeting event is associated with opposite genotypes in the MCF-7 and CAMA1 cells. Therefore, the observation of concordant changes in the two different cell lines supports these being bona fide targets affected by *GATA3* mutation, rather than effects of the gene targeting process. To further validate these target genes, we re-expressed the Gata3 D336Gfs*17 mutant in the two MCF-7-derived *GATA3* WT clones (Figure 6A). Truncated mutant Gata3 protein was expressed at a similar level to that in MCF-7 cells. Re-expression of mutant Gata3 induced gene expression changes concordant with MCF-7, suggesting that some of these changes are at least partly due to gain-of-function of the mutant protein (Figure 6B). Addback of mutant GATA3 also enhanced soft agar colony formation in both wild type clones, again consistent with a dominant gain of function for the mutant protein (*p* = 0.003 by *t* test, Figure 6C).

### Mutant Gata3 enhances breast cancer tumor growth *in vivo*

**Figure 7:**
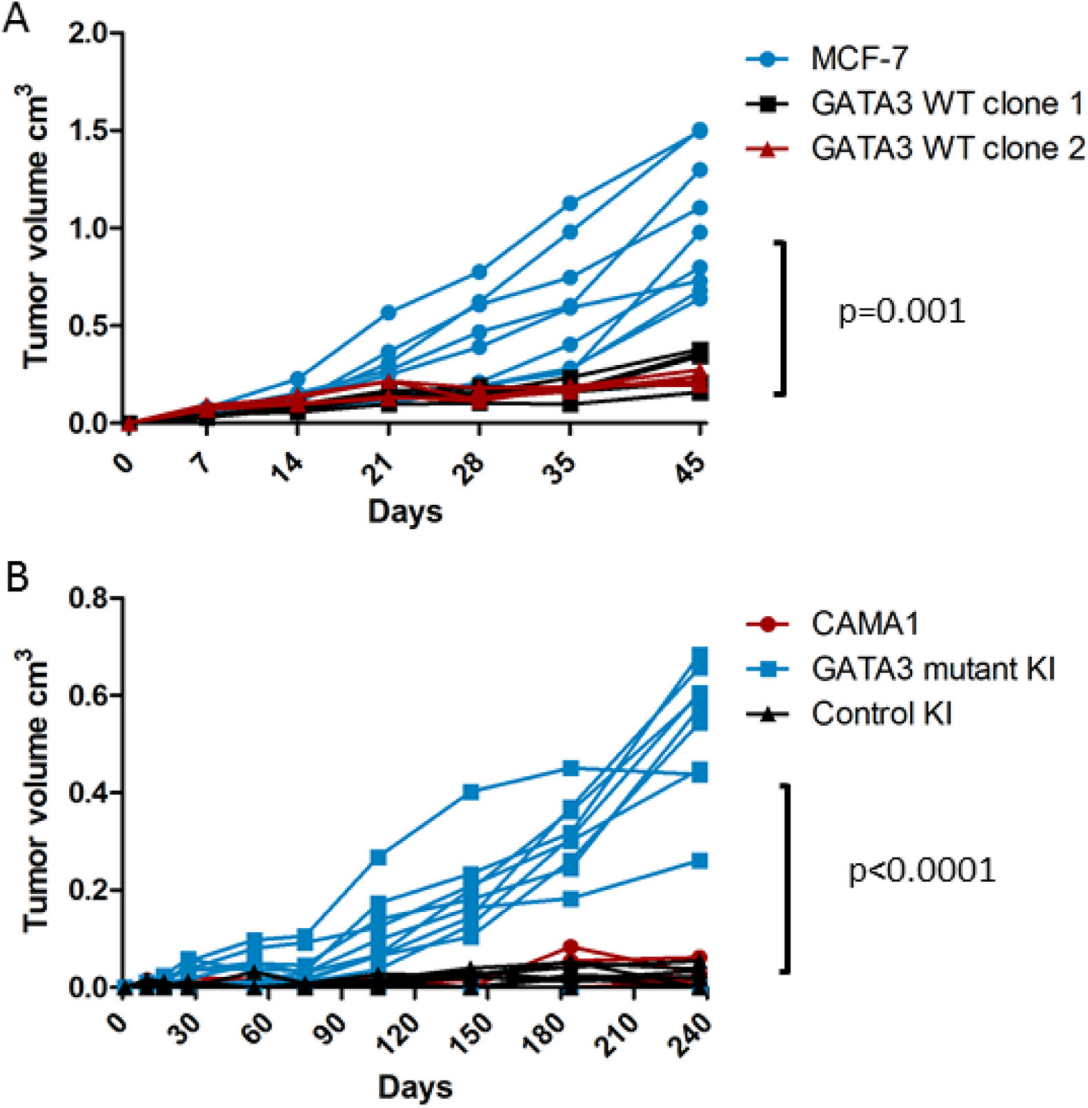
Xenograft growth of isogenic GATA3 mutant and wild type breast cancer cell lines (**A**) Nude mouse xenograft growth of MCF-7 (*n* = 9) and GATA3 wild type derivative clones (*n* = 5 each). *P* value for repeated measures two-way ANOVA is shown. (**B**) NSG mouse xenograft growth of CAMA1, GATA3 mutant knock-in, and control knock-in cells (*n* = 10 mice per group). *P* value for repeated measures two-way ANOVA is shown.

Parental MCF-7 cells formed tumors in estrogen-supplemented nude mice, as expected. Tumors did not form in the absence of estrogen pellet implantation (data not shown), confirming that these cells had not acquired estrogen-independence. *GATA3* wild type cells (*n* = 5 per clone) grew significantly more slowly and formed smaller tumors compared to parental MCF-7 (*n* = 9, *p* = 0.001 by repeated measures two-way ANOVA, Figure 7A). Parental CAMA1 cells and *GATA3* wild type-expressing control knock-in cells failed to grow after orthotopic implantation in NSG mice, but the *GATA3*-mutant-expressing targeted clone did form tumors (*n* = 10 per group, *p* < 0.0001 by repeated measures two-way ANOVA, Figure 7B). Thus, correction of mutant GATA3 back to wild type impaired estrogen-dependent growth of MCF-7 breast cancer cells *in vivo*, and knock-in mutant GATA3 was sufficient to confer the ability of CAMA1 cells to grow as xenografts in immunocompromised mice.

## DISCUSSION

Gata3 is essential for breast development [27, 28]. Heterozygous mutations in *GATA3*, mostly frameshifts, occur in approximately 15% of primary ER+ human breast cancers, suggesting that Gata3 may function as a haploinsufficient tumor suppressor for luminal breast cancers. However, clustering of mutations toward the C-terminal region of the protein and continued high expression of the mutant protein argue against a simple loss of function effect. Previous work using ectopic overexpression of Gata3 in breast cancer cell lines that are ER and Gata3-negative, such as MDA-MB-231, has demonstrated the ability of ectopic Gata3 expression to suppress tumor and metastasis formation, although the relevance of manipulating Gata3 in an ER-negative, non-luminal cell type is uncertain [17–21, 29]. Moreover, some studies of Gata3 function have used MCF-7 cells but have not generally considered the fact that a GATA3 mutation is present in this cell line. Knockdown of Gata3 in MCF-7 cells reduces both wild type and mutant proteins and therefore may not be a surrogate for the effects of *GATA3* mutations. In this study, we have used gene editing to examine the functional consequences of a recurrent *GATA3* truncating mutation in two ER+ breast cancer cell lines, including MCF-7, where the mutation naturally occurs.

A caveat of our study is that we have modeled the effects of a specific ZF2 frameshift mutation. We and other investigators have noted that *GATA3* mutations cluster in two protein regions: ZF2 and the C-terminus. Whereas ZF2 mutations are truncating, many of the C-terminal mutations occur very close to the end of the protein and most of these shift into the same +1 reading frame to cause the addition of a neo-peptide sequence after the frameshift, resulting in a protein of higher predicted molecular weight [3, 30]. It is less clear how C-terminal *GATA3* mutations would impact protein function, but, as suggested by others, there could be distinct effects of these two mutation classes [30]. Our ability to model these C-terminal mutations in a MCF-7 *GATA3* WT background may provide a more physiologic context to study their function.

The transcriptional effects of Gata3 are complex, as Gata3 has been documented to bind to thousands of sites in the genome and to be associated with multiple other transcription factors, including ERα, TCF7L2, RARA, and FOXA1 [12, 19, 31, 32]. These relationships are also affected by feedback loops and transcriptional autoregulation [11]. *In vitro* evidence suggests that the ZF2 mutations in Gata3 are loss of function—at least at the level of DNA binding—although some CHIP-seq data show that DNA-binding-defective Gata3 mutants can still be recruited to a subset of genomic binding sites, perhaps through protein-protein interactions [3, 25, 33]. By using gene editing, we have simultaneously reduced dosage of a wild type allele when we have added a mutant allele, and vice-versa. This makes mechanistic separation of haploinsufficiency and gain-of-function effects difficult, although our approach faithfully models the effects of mutations that occur in human breast cancers. Our microarray data identifies a set of genes which are concordantly affected by *GATA3* mutation in two ER+ breast cancer cell lines. We also observed concordant differential expression for a subset of these genes according to *GATA3* mutation status in primary ER+ breast cancers from the TCGA dataset. We observed both upregulated and downregulated genes, consistent with both transcriptional activation and repression mediated by Gata3. Add-back of truncated mutant Gata3 recapitulated some of these transcriptional effects, consistent with at least a partial gain-of-function or dominant-negative effect.

The biological relevance of the genes affected by *GATA3* mutation in our study will require further analysis, but several of them have plausible roles in breast cancer. CEACAM6 has been shown to be a marker of atypical ductal hyperplasia lesions at increased risk of progressing to invasive breast cancer, and its expression is highest in ER+ and Her2+ breast cancers [34, 35]. High CEACAM6 expression was associated with breast cancer recurrence following adjuvant tamoxifen, and knockdown of CEACAM6 restored tamoxifen sensitivity and reduced clonogenic growth, migration, and invasion of tamoxifen-resistant MCF-7 cells [36, 37]. *PPT1* encodes a palmitoyl-protein thioesterase, which removes the post-translational modification palmitate from proteins, although the spectrum of its substrates is unknown. Intriguingly, palmitoylation of ERα has been shown to mediate its membrane localization and mitogenic function [38, 39]. Expression of TBC1D9, a Rab GTPase accelerating protein, is highly correlated with ERα and Gata3 expression in human breast cancers [40].

It is likely that *GATA3* mutation affects developmental processes involving mammary cell lineage determination, which are sensitive to *GATA3* dosage [27, 28, 30, 41, 42]. Mice with mammary gland-specific deletion of *GATA3* have defective luminal differentiation [27, 28]. A subset of Gata3 regulated genes may be critical for cell fate in the mammary gland, and dysregulation of transcription through *GATA3* mutation may block terminal differentiation and start cells on the road to cancer. Hemizygous deletion of *GATA3* in the mammary gland accelerates tumor formation in the PyMT model, and transgenic overexpression of wild type Gata3 delays tumor formation [41]. An early, developmental role for *GATA3* mutation is not addressed by our cancer cell line model, but an early role does not necessarily preclude a later impact of these mutations on breast cancer phenotypes and outcomes. Nonetheless, we did not see significant effects on many of the previously reported Gata3 target genes in our isogenic cell line models. It is possible that a different subset of genes is affected by *GATA3* mutation in luminal progenitor cells than in established breast cancer cells.

It has long been recognized that *GATA3* mutations occur in the better-prognosis luminal subtypes of breast cancer. Recent data from the METABRIC project show improved breast cancer-specific survival for patients with ER+ tumors with *GATA3* mutations, although this conclusion is not universally supported by mutation prevalence in metastatic breast cancer [1, 10, 43]. We sought to determine whether *GATA3* mutation affects sensitivity to endocrine therapy or chemotherapy, but we did not find significant differences in response to tamoxifen, fulvestrant, paclitaxel, or doxorubicin in our MCF-7 cell line panel. *GATA3* mutant MCF-7 cells did show an enhanced ability to form anchorage-independent colonies under estrogen-free conditions, but they were unable to grow as xenografts in non-estrogenized mice. Thus, *GATA3* mutation does not cause the estrogen-independent phenotype of long term estrogen-deprived cells, which has been used as a model for aromatase inhibitor resistance. Our results are consistent with clinical trial results showing that *GATA3* mutations did not impact response rates to neoadjuvant aromatase inhibitor therapy [44]. *GATA3* WT MCF-7 and CAMA1 cells grew poorly as xenografts in immunocompromised mice compared to *GATA3* mutant cells, however, raising the possibility that *GATA3* mutant breast cancer cells do depend on the mutant protein for tumor growth *in vivo*. Whether this property can be attributed to specific genes regulated by Gata3 will require further study. Therefore, whether or not *GATA3* mutant breast cancers have better clinical outcomes than ER+ *GATA3* wild type tumors, it remains possible that mutant Gata3 or its downstream effectors could be rational targets for breast cancer therapy.

## MATERIALS AND METHODS

### Cell lines

MCF-7 and CAMA1 cells were originally obtained from ATCC and the identity of the parental cell lines and the gene targeted derivatives was verified in October 2016 by STR analysis at the Johns Hopkins Genetic Resource Core Facility. Cells were maintained in DMEM (Cellgro) supplemented with 5% (MCF-7) or 10% (CAMA1) FBS (Hyclone) and 100 U/mL penicillin and 100 µg/mL streptomycin (Cellgro). For growth assays the following media formulations were used: phenol-red free DMEM/F12 (Invitrogen) with 0.5% charcoal-dextran treated FBS (Hyclone) with or without 1 nM 17-β-estradiol (Sigma). All cells were cultured at 37°C at 5% CO_2_.

### Gene targeting at the *GATA3* locus

Gene targeting was conducted with recombinant adeno-associated viral (rAAV) vectors as described [24, 45, 46]. 5′- and 3′-homology arms were constructed by high-fidelity PCR using genomic DNA (gDNA) from MCF10A as template for the homology arms. To correct the *GATA3* c1006_1007insG mutation back to wild type, two rounds of gene targeting were employed. For the first round, *GATA3* exon 5 was included in the 5′ homology arm. Cells with one corrected *GATA3* allele were then subjected to a second round of gene targeting using a different construct with the wild type exon 5 located in the 3’ homology arm. CAMA1 cells were targeted as above using a construct with the *GATA3* c1006_1007insG mutation in exon 5 in the 5′ homology arm, which was generated by PCR using MCF-7 genomic DNA as a template. Primer sequences for PCR are available on request.

### DNA and RNA extraction, cDNA synthesis, PCR, and sequencing

Genomic DNA and total RNA were prepared from cells using QIAamp DNA Blood kits and RNeasy kits (Qiagen), respectively. RNA was treated on-column with DNAaseI. CDNA was synthesized with First-Strand cDNA Synthesis kit (GE Biosciences). PCR amplification was done using GeneAmp 9700 (Applied Biosystems) and Phusion-HF (NEB) or Platinum Taq polymerase (Invitrogen). PCR primers to amplify cDNA were designed with forward and reverse primers located in distinct exons. Quantitative reverse transcriptase PCR was performed using SYBR green on a BioRad iCycler. Relative gene expression in triplicate wells was quantified using the ∆∆Ct method with TATA binding protein (TBP) as a reference. Automated direct sequencing of PCR products was carried out by the Johns Hopkins DNA Synthesis and Sequencing Facility. Primer sequences for PCR and direct sequencing are available on request.

### Re-expression of mutant Gata3

Full length cDNA encoding the Gata3 D336Gfs*17 protein was cloned into pIRESneo3 (Clontech). MCF-7 *GATA3* WT clones were transfected with pIRESneo3 or pIRESneo3/Gata3 D336fs*17 using FuGENE 6 (Promega) and selected with G418 as stable pools.

### Soft agar colony formation

3 × 10^4^ exponentially growing cells were cast in 3 mL of top layer medium composed of supplemented phenol red free DMEM/F12 and 0.4% UltraPure agarose (Invitrogen) and poured on top of a 2 mL bottom layer containing 0.6% agarose in six-well tissue culture plates. Supplements consisted of 0.5% CD-FBS plus 1 nM 17-β-estradiol or 0.5% CD-FBS alone. Supplemented DMEM/F12 was added to the wells twice a week. Two independent experiments were done in triplicate. Colonies were fixed and stained with 0.05% crystal violet in 10% ethanol. For the experiment in Figure 2, stained plates were scanned and macroscopically visible colonies were counted using ImageJ. For the experiment in Figure 6, media condition was 0.5% CD-FBS without estrogen. Four images of each well were captured using a dissecting microscope at 10X magnification, and colonies with pixel size > 200 were quantified using ImageJ and averaged for each well. Triplicate wells for each sample were then averaged.

### Drug treatment experiments

Cells were plated in triplicate wells. For experiments using 4-hydroxy tamoxifen and fulvestrant, cells were cultured in medium with 1 nM 17-β-estradiol as described above. Other experiments were conducted in DMEM/5% FBS medium. Drugs were added on day 1. Cells were harvested and Trypan blue-excluding cells were counted on the indicated days. Viable cell numbers were normalized to day 1 values for each sample. Viability versus log of the drug concentration was plotted and fit with a non-linear regression model with variable slope to calculate IC^50^ values using GraphPad Prism 5. For each drug, curves for MCF-7 were compared with curves for the average of the two GATA3 wild type clones using extra sum-of-squares F test. The *p* value threshold of 0.05 was adjusted for multiple comparisons.

### Immunoblotting

Whole-cell protein extracts prepared in Laemmli sample buffer were resolved by SDS-PAGE using NuPage gels (Invitrogen), transferred to Invitrolon polyvinylidene difluoride membranes (Invitrogen), and probed with primary and horseradish peroxidase–conjugated secondary antibodies. Primary antibodies were anti-Gata3 mouse monoclonal antibody (Sc-269, Santa Cruz Biotechnology), anti-ERα mouse monoclonal antibody (Beckman Coulter), and anti–glyceraldehyde-3-phosphate dehydrogenase (GAPDH) mouse monoclonal antibody (6C5; Abcam). Blots were exposed to Kodak XAR film using chemiluminescence for detection (Perkin Elmer).

### Xenograft experiments

All experiments were conducted under a protocol approved by the institutional animal care and use committee and followed the National Institutes of Health Guide for the Care and Use of Laboratory Animals. For experiments with MCF-7 and its derivatives, 8- to 10-week-old female athymic nude mice (Taconic) were used. Two days prior to cell inoculation, mice were surgically implanted with slow-release 17-β-estradiol pellets. For each group, five (each *GATA3* WT clone) or nine (MCF-7) mice were injected subcutaneously the flank with 200 μl mixture containing 1 × 10^6^ cells in 20% PBS and 80% Matrigel. For CAMA1 cells, NSG mice were injected in the mammary fat pad with 2 × 10^6^ cells in 20% PBS and 80% Matrigel, 10 mice per group. After the appearance of palpable tumors, tumors were measured with the frequency shown and volumes were calculated by multiplying length, width, and height for each individual tumor.

### Microarray gene expression analysis

All cell lines were cultured in 0.5% CD-FBS medium without estrogen prior to RNA harvest. Three separate microarray hybridizations were run, comprising a total of four biological replicates of parental MCF-7 cells, five biological replicates of MCF-7 GATA3 wild type clone 1, and two biological replicates of MCF-7 GATA3 wild type clone 2. Single samples from parental CAMA1 and GATA3 mutant knock-in CAMA1 cells were hybridized. Sample quality assessment and microarray analysis using HumanHT-12 v4 Expression BeadChip arrays (Illumina, San Diego, CA) were performed at the Sidney Kimmel Comprehensive Cancer Center Microarray Core Facility at Johns Hopkins University, Baltimore. 500 ng total RNA from each sample was amplified and labeled using the Illumina TotalPrep RNA Amplification Kit (AMIL1791, Ambion, Austin, TX) as described in the instruction manual. 750 ng biotin-labeled cRNA was combined with hybridization buffer and hybridized to the array at 58°C for 16–20 hours. After hybridization, the hybridization cartridge was disassembled and the array was washed with buffer at 55°C and blocked at room temperature. Bound biotinylated cRNA was stained with streptavidin-Cy3 and then washed. Dried arrays were stored in a dark box until scanned with iScan System. Data were extracted with Gene Expression Module in GenomeStudio Software. Quantile normalization across all arrays was performed using the lumi package in R, and differential gene expression in MCF-7 was calculated with the R package limma using a Benjamini-Hochberg adjusted *p* value of < 0.05. Differential gene expression between CAMA1 parental and mutant knock-in clones was performed using BRB-ArrayTools v4.2 with a fold-change cut-off of 1.25 [47]. Raw and normalized gene expression data have been submitted to the NCBI Gene Expression Omnibus under accession number GSE101780.

### TCGA RNAseq analysis

TCGA breast cancer data was analyzed using CBioPortal [48]. 594 ER+ cases with RNAseq data were divided into two groups based on presence (86 cases) or absence (508 cases) of *GATA3* mutations. Differentially expressed transcripts were identified with a Benjamini-Hochberg *q* value < 0.01. Gene list overlap significance testing was carried out using hypergeometric probability test.

### Statistical analysis

Cell proliferation assays and xenograft growth experiments were compared using repeated measures two-way ANOVA with Bonferroni adjustment for multiple comparisons. Colony formation was compared using unpaired *t* tests. All analyses were conducted using GraphPad Prism 5 software.

## SUPPLEMENTARY MATERIALS TABLE




