# Supplementary material for: GATA3 frameshift mutation promotes tumor growth in human luminal breast cancer cells and induces transcriptional changes seen in primary GATA3 mutant breast cancers
Source: Oncotarget. 2017 Oct 20;8(61):103415–27. doi: 10.18632/oncotarget.21910 (PMC5732738; doi:10.18632/oncotarget.21910)
Supplement: Supplementary file 1 [file oncotarget-08-103415-s001.pdf]

GATA3 frameshift mutation promotes tumor growth in human luminal breast cancer cells and induces transcriptional changes seen in primary *GATA3* mutant breast cancers

SUPPLEMENTARY MATERIALS

Supplementary Table 1: Genes with significantly different expression between parental MCF-7 and GATA3 WT clones with adjusted *P* value < 0.05. See Supplementary_Table_1
